# Determinants of the intention to use e-Health by community dwelling older people

**DOI:** 10.1186/s12913-015-0765-8

**Published:** 2015-03-15

**Authors:** Anke J E de Veer, José M Peeters, Anne EM Brabers, Francois G Schellevis, Jany JD JM Rademakers, Anneke L Francke

**Affiliations:** NIVEL, Netherlands Institute for Health Services Research, PO box 1568, 3500 BN Utrecht, The Netherlands; Department of General Practice and Elderly Care Medicine/EMGO Institute for Health and Care Research, VU University Medical Center, Amsterdam, The Netherlands; Department of Public and Occupational Health, EMGO Institute for Health and Care Research, VU University Medical Center, Amsterdam, The Netherlands; Expertise Center for Palliative Care Amsterdam, VU University Medical Center, Amsterdam, The Netherlands

**Keywords:** Technology acceptance, e-Health, UTAUT, Community, Older people

## Abstract

**Background:**

In the future, an increasing number of elderly people will be asked to accept care delivered through the Internet. For example, health-care professionals can provide treatment or support via telecare. But do elderly people intend to use such so-called e-Health applications? The objective of this study is to gain insight into the intention of older people, i.e. the elderly of the future, to use e-Health applications. Using elements of the Unified Theory of Acceptance and Use of Technology (UTAUT), we hypothesized that their intention is related to the belief that e-Health will help (performance expectancy), the perceived ease of use (effort expectancy), the beliefs of important others (social influence), and the self-efficacy concerning Internet usage.

**Methods:**

A pre-structured questionnaire was completed by 1014 people aged between 57 and 77 (response 67%). The hypothesized relationships were tested using nested linear regression analyses.

**Results:**

If offered an e-Health application in the future, 63.1% of the respondents would definitely or probably use it. In general, people with a lower level of education had less intention of using e-Health. The majority of respondents perceived e-Health as easy to use (60.8%) and easy to learn (68.4%), items that constitute the scale for effort expectancy. Items in the performance expectancy scale generally scored lower: 45.8% perceived e-Health as useful and 38.2% perceived it as a pleasant way to interact. The tested model showed that expected performance and effort were highly related to intention to use e-Health. In addition, self-efficacy was related to intention to use while social influence was not.

**Conclusions:**

Acceptance of e-Health can be increased by informing people about the potential benefits of e-Health and letting them practice with the application. Special attention should be paid to people with less education and people who have not used the Internet before.

**Electronic supplementary material:**

The online version of this article (doi:10.1186/s12913-015-0765-8) contains supplementary material, which is available to authorized users.

## Background

Due to reforms in long-term care, people are now expected to live on their own (rather than in care homes) for as long as possible. E-Health applications are considered to be important tools in achieving this goal, together with increased support by family, friends, and volunteers. E-Health is seen as an emerging field in the intersection of medical informatics, public health, and business, and refers to health services and information delivered or enhanced through the Internet and related technologies [1]. In this article, we focus on health care delivered through the Internet. Examples are the exchange of information between patients and professionals through the Internet and the provision of support by professionals through telecare. E-Health applications have the potential to support self-management and self-care [2,3], and to reduce the costs of care [4,5] for community-dwelling older people. However, evidence regarding these effects is still scarce, due either to flaws in the study design [6] or insufficient implementation [7]. One of the factors impeding implementation might be that the target group has no intention of using e-Health. Knowledge about what influences the intentions to use e-Health can help select effective implementation strategies.

The Netherlands is one of the European Union countries with the most highly developed digital infrastructure and highest proportion of households with Internet access [8]. The use of the Internet has expanded rapidly in the Netherlands. These days, 80% of 65–75 year olds have used the Internet at least once [9]. Between 2005 and 2013, the percentage of 65–75 year olds who use the Internet daily increased from 15% to 55% [9]. So Internet access is not an important factor impeding the use e-Health, although familiarity might still play a role for some people.

There are only a few quantitative studies of the intention to use e-Health and/or acceptance of e-Health among community-dwelling older people. A systematic review of studies of the acceptance of technology for supporting aging at home identified sixteen studies, of which only three had a quantitative design [10]. The authors concluded that acceptance of technology is influenced by six themes: concerns regarding technology (e.g. high costs, privacy implications, and usability factors), the expected benefits of technology (e.g. increased safety and perceived usefulness), the need for technology (e.g. perceived need and subjective health status), alternatives to technology (e.g. help from family or spouse), social influences (e.g. the influence of family, friends, and professional caregivers), and characteristics of the older people (e.g. the desire to grow old at home). Fourteen out of the sixteen studies did not use an existing framework or model to analyze technology acceptance. It was concluded that further research is needed to determine how the aforementioned factors are interrelated and how they relate to existing models of technology acceptance.

One of these models is the Unified Theory of Acceptance and Use of Technology (UTAUT), a model designed to explain the intention to use a technology. The UTAUT model is an extension of the Technology Acceptance Model (TAM), a widely recognized model to explain intention to use in numerous industries [11]. UTAUT, developed by Venkatesh et al. [12], is based on the comparison of eight theories concerning determinants of acceptance. These theories included Fishbein and Ajzen’s Theory of Reasoned Action, Rogers’s Innovation Diffusion Theory, and Davis’s Technology Acceptance Model. According to UTAUT, four main factors influence intention to use, whereby intention to use is defined as the degree to which a person has formulated conscious plans to perform or not perform some specified future behavior [12]. The factors are:

1. Performance expectancy. The degree to which an individual believes that using the system will help him or her (also referred to as “perceived usefulness” in TAM);
2. Effort expectancy. The degree of ease associated with the use of the system (also referred to as “ease of use” in TAM);
3. Social influence. The degree to which an individual perceives that important others believe he or she should use the new system;
4. Facilitating conditions. The degree to which an individual believes that an organizational and technical infrastructure exists to support use of the system. Internet efficacy has been identified as a facilitating factor predicting the use of e-Health [13,14].

UTAUT and TAM have mainly been tested in the context of health care and among health-care professionals [11,14,15] and patients [13,16], while they have not been tested nearly as much among (healthy) older people. Our study explores the determinants of the intention to use e-Health among ‘older people’, that is people between 57 and 77 years old who will form the next generation of the elderly. Studies predicting acceptance of a fitness program and health information online among relatively healthy community-dwelling older people showed that performance and effort expectancy were relevant predictors of acceptance [17,18]. Acceptance of everyday technologies such as mobile phones and remote control devices should also include individual characteristics such as age, sex, and education [19].

Although it is a health-care policy aim in the Netherlands and many other countries to increase the use of e-Health, knowledge about the intention to use e-Health within the population of community-dwelling older people is still scarce. Therefore, the research objectives of this study were (1) to identify (groups of) community-dwelling older people who are open to the use of e-Health and (2) to explore the beliefs of community-dwelling older people related to their intention to use e-Health, by using the UTAUT model. Three research questions were formulated:

1.
  - Do community-dwelling older people intend to use e-Health?
  - Are age, sex, and education related to intention to use?
2. What do community-dwelling older people perceive to be the characteristics of e-Health?
3. To what extent is the intention to use e-Health related to performance expectancy, effort expectancy, social influence, and self-efficacy concerning Internet usage?

## Methods

### Study population

The study population for the present study consisted of a sample from the Dutch Health Care Consumer Panel run by NIVEL, Netherlands Institute for Health Services Research. This panel consisted at the time of this study (May, 2013) of about 6,000 people aged 18 and older [20]. From the panel, samples can be drawn that are representative of the general Dutch population in terms of age and sex. In addition, it is possible to draw a sample that is based on a selection of background characteristics that we know from our panel members. It is not possible for consumers to sign up for the panel on their own initiative. The panel is refreshed on a regular basis to ensure that members do not develop specific knowledge of, or interest in, health-care issues, and that no questionnaire fatigue occurs. New members of the panel are sampled from the general population.

A stratified random sample was drawn of 750 persons aged between 57 and 66, and 750 persons aged between 67 and 77. The sample was stratified to ensure that we had enough people in the highest age categories in the response group. The 1500 persons received a questionnaire by post or by e-mail the Internet according to their previously stated preference.

### Ethical statement

The database of the addresses is registered with the Dutch Data Protection Authority (no. 1262949, see http://www.cbpweb.nl for more information). In addition, the Consumer Panel has a privacy policy. All respondents received an information letter about the aim and goal of the study along with the survey questionnaire. The responses were stored and analysed anonymously, in accordance with the Dutch Personal Data Protection Act (http://www.privacy.nl/uploads/guide_for_controller_ministry_justice.pdf). Formal ethical approval of this study was not required under the applicable Dutch legislation (http://www.ccmo.nl/en/), since all respondents were competent individuals and this survey study did not involve any interventions or treatments.

### Questionnaire

The questionnaire surveyed the respondents’ wishes concerning future care and housing. Among other subjects, the questionnaire addressed e-Health issues. This part of the questionnaire had already been successfully used in previous research [21], in which the questions on performance and effort expectancy were derived from Venkatesh et al. [12].

The questions on e-Health issues started with a description of e-Health, followed by pre-structured, multiple-choice questions (see Additional file 1). E-Health was described as care through the Internet such as:

- Making an appointment with a health-care professional;
- Asking a health-care professional a question;
- Getting treatment or support via telecare from a health-care professional;
- Measuring your weight, blood pressure or blood sugar level, for example, at home and sending the measurement to your health-care professional.

The Internet can be used on all kind of devices, such as a computer, tablet or smartphone. Besides these Internet contacts, you would still have face-to-face contacts with the health-care professional.

Specific questions covered the following:

- Intention to use e-Health. Respondents were asked “Do you think you would use one of the above-mentioned Internet applications in the future if you were offered the opportunity?” Possible answers were “yes, definitely” (score = 5), “yes, probably” (score = 4), “I don’t know yet” (score = 3), “no, probably not” (score = 2), “no, definitely not” (score = 1).
- Performance expectancy. Respondents were asked to rate the following four statements: “Contacting health-care professionals via the Internet (1) makes it easier to contact a health-care professional when I want, (2) enables me to live independently for longer, (3) works well, (4) is a pleasant way to interact”. The possible answers were “strongly agree” (score = 5), “agree” (score = 4), “I don’t know” (score = 3), “disagree” (score = 2), “strongly disagree” (score = 1). The score for performance expectancy was the average of the scores on the four items. Cronbach’s alpha was .79.
- Effort expectancy. Three statements were used: “Contacting health-care professionals via the Internet (1) is easy to learn, (2) fits easily into my daily routine, (3) is easy to do.” The categories were the same as that of performance expectancy. The score for effort expectancy was the average of the scores on the three items. Cronbach’s alpha was .84.
- Social influence. The statement was “Contacting health-care professionals via the Internet is something my family or friends would like to do”. The same answer categories were given as for performance expectancy.
- Self-efficacy. Self-efficacy refers to the person’s belief that he or she is able to successfully use the Internet. The question was: “How easy or difficult do you find it to use the Internet”. The possible answers were “very difficult” (score = 1), “difficult”(score = 2), “neutral” (score = 3), “easy” (score = 4), “very easy” (score = 5), “I don ‘t know; I don’t use the Internet”. The final answer was used to construct the variable “Internet user”. The score on this variable was “yes” (score = 1) if the respondent rated their self-efficacy and “no” (score = 0) if the respondent gave the final answer “I don ‘t know; I don’t use the Internet”.

Age, sex, and education (low, medium, high) were included as background variables to explore which sociodemographic groups intend to use e-Health applications and which are less inclined to do so.

### Statistical analyses

The first and second research questions were answered using descriptive statistics. Because we made use of a stratified sample with equal numbers of participants in the two age groups, we weighted the descriptive analysis for age and sex in such a way that the results reflected the distribution of age and sex within the population of Dutch 57 to 77 year olds (based on data from Statistics Netherlands). Subgroup analyses were performed to unravel the interactions between the respondents’ background characteristics and their intention to use e-Health. In each subgroup, the relationship between one background characteristic and the intention to use e-Health was examined, keeping the combination of other background characteristics constant.

**Figure 1:**
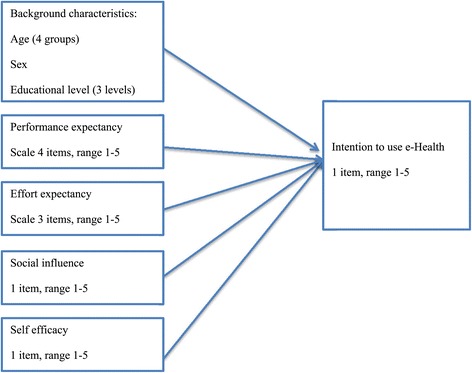
**The model tested to explain intention to use e-Health, derived from UTAUT.**

To analyze the relationship between the intention to use e-Health and the factors in the UTAUT model, we conducted a nested linear regression analysis, starting with the background variables (age, sex, and education) and then adding performance expectancy, effort expectancy, social influence, and self-efficacy blockwise to the model (Figure 1). People who had never used the Internet had difficulties answering the questions on performance expectancy, effort expectancy, social influence, and self-efficacy: 62.7%-76.7% answered “don’t know”. The respondents who did not have any experience with the Internet were therefore left out of the regression analyses that tested the model. All analyses were performed using STATA 13.0.

## Results

### Respondents

A total of 1014 respondents completed the questionnaire (response 66.7%; 496 women and 518 men). The response of the 67 to 77 year olds was higher (n = 542, 72.3%) than the response of the 57 to 66 year olds (n = 472, 62.9%). One fifth (21.3%) of the respondents had a low level of education (none, primary school or prevocational education), 54.8% had a medium educational level (secondary or vocational education), 20.3% a high educational level (higher education or university), and 3.6% unknown.

Weighted for age and sex, 8.2% of the 57 to 77 year olds did not have any experience with the Internet. This group, referred to as “non-users”, had different background characteristics and answering patterns compared with the group who used the Internet (p < .05). The non-users were older than the users (53.3% versus 25.3% were 72 to 77 years old). Also half of the non-users (50.6%) had a low educational level and only 5.6% were highly educated. Of the users, 18.9% had a low educational level and 22.7% were highly educated (p < .05). No statistically significant relationship was found between Internet use and sex (p = .48).

### Intention to use e-Health

As shown in Table 1, 63.1% of the community-dwelling older people intended to use e-Health if offered the opportunity. On the other hand, 15.9% thought they would refuse it. There were statistically significant bivariate relationships with age, sex, educational background, and status as an Internet user (Table 2). Nearly seventy percent of the 57–61 year olds (68.2%) and 62–66 year olds (69.9%) probably or definitely intended to use e-Health when offered, whereas half (52.2%) of 72–77 year olds intended to do so. Of the men, 70.7% probably or definitely intended to use e-Health, whereas 55.9% of the women intended to do so. Education had an effect too: 75.0% of respondents with a high level of education intended to use e-Health, whereas only 42.7% of those with a low level of education intended to do so. Nearly two-thirds of the non-Internet-users (64.7%) did not intend to use e-Health applications if asked to use them; 30.2% were determined not to use them and 34.5% said they probably would not use them.

**Table 1:** **Intention to use e-Health (N = 982)**

**Do you think you would use one of the above-mentioned Internet applications in the future if you were offered the opportunity**	**%(1)**
- Yes, definitely (value = 5)	31.4
- Yes, probably (value = 4)	31.7
- I don’t know yet (value = 3)	21.0
- Probably not (value = 2)	10.0
- Definitely not (value = 1)	5.9

(1)Percentages are weighted by age and sex.

**Table 2:** **Intention to use e-Health and bivariate relationships with background characteristics**

**Background characteristics**	**Yes, definitely** **%(1)**	**Yes, probably** **%**	**Don’t know** **%**	**Probably not** **%**	**Definitely not** **%**	**Chi2**	**p-value**
Age							
- 57-61	39.6	28.6	17.9	7.9	6.0	38.35	P < .001
- 62-66	33.1	36.8	18.6	7.3	4.2		
- 67-71	25.9	30.2	26.5	12.9	4.4		
- 72-77	23.5	28.7	23.6	14.3	9.9		
Sex							
- Male	36.4	34.3	14.9	8.9	5.5	23.28	p < .001
- Female	26.7	29.2	26.8	11.0	6.3		
Educational level							
- Low	20.3	22.4	27.7	16.2	13.4	63.7	P < .001
- Medium	30.1	34.8	21.6	8.6	4.8		
- High	44.5	30.5	14.4	7.9	2.7		
Internet user							
- Yes	33.5	34.0	20.9	7.9	3.7	197.0	P < .001
- No	6.2	6.5	22.6	34.5	30.2		

(1)Percentages are weighted by age and sex.

Due to rounding, percentages do not always add up to 100.0%.

Subgroup analyses showed relatively high percentages (that is between 33% and 40%) of people who probably or definitely intended *not* to use e-Health among the following groups: women aged between 57 and 61 with a low level of education and men aged between 62 and 77 with a low level of education.

### Perceptions of e-Health

Table 3 shows how older people perceived e-Health. In general, the aspects perceived in the most favorable light concerned the effort needed to use these applications. The majority (60.8-68.4%) thought it would be easy to use the applications, whereas 12.4-17.6% thought it would not.

**Table 3:** **Perceptions of older people concerning the use of e-Health applications(1)**

**Contacting health-care professionals via the Internet ….**	**(Strongly) disagree** **%**	**Don’t know** **%**	**(Strongly) agree** **%**
*Performance expectancy*			
- Makes it easier to contact a health-care professional when I want	12.2	19.6	68.2
- Enables me to live independently for longer	12.8	29.8	57.4
- Works well	15.9	38.3	45.8
- Is a pleasant way to interact with health-care professionals	37.1	24.7	38.2
*Effort expectancy*			
- Is easy to learn	15.5	16.2	68.4
- Fits easily into my daily routine	12.4	23.1	64.5
- Is easy to do	17.6	21.6	60.8
*Social influence*			
- Is something my family or friends would like to do	11.4	43.0	45.5
*Self-efficacy*	(Very) difficult	Neutral	(Very) easy
- How easy or difficult do you find is it to use the Internet?(2)	15.2	36.2	48.6

(1)Weighted by age and sex.

(2)Only answered by respondents who use or have used the Internet.

Due to rounding percentages do not always add up to 100.0%.

The majority agreed with the statement that e-Health applications would make it easier to contact health-care professionals and to live independently for longer. However only a minority agreed that these applications work well (45.8%) and are a pleasant way to interact (38.2%). A considerable proportion (37.1%) disagreed that e-Health applications offer a pleasant way to interact.

Self-efficacy varied: 48.6% found the Internet easy or very easy and 15.2% found the Internet difficult or very difficult.

### Determinants of intention to use

Correlations (not in table) between intention to use, performance expectancy, effort expectancy, social influence, and self-efficacy were moderately strong (all Pearson r values between .36 and .69, p < .001), except for the correlations of social influence with intention to use (r = .25, p < .001) and with self-efficacy (r = .17, p < .001). Self-efficacy was related most strongly to effort expectancy (r = .62). In the first regression analysis (Table 4), the background characteristics only explained 6% of the variation in the intention to use e-Health. The standardized regression coefficients (Betas) for sex and education changed when the other variables were added, finally losing statistical significance in the full regression model.

**Table 4:** **Nested regression analysis to explain intention to use e-Health (n = 834)**

	**Block 1**	**Block 2**	**Block 3**	**Block 4**	**Final model**
**R** ^**2**^	**.06**	**.32**	**.40**	**.40**	**.41**
**Change in R** ^**2**^		**.26**	**.08**	**.00**	**.01**
**Sign of R** ^**2**^ **change**	**<.001**	**<.001**	**<.001**	**.463**	**.001**
**Independent variables added**	***Beta***	***Beta***	***Beta***	***Beta***	***Beta***
Age					
- 57–61 (ref)					
- 62-66					
- 67-71					
- 72-77		-.06*			
Sex					
- Male (ref)					
- Female	-.14***	-.06**	-.05*	-.05*	
Educational level					
- High (ref)					
- Medium	.	-.08**			
- Low	-.20***	-.16***	-.06*	-.06*	.
Performance expectancy		.52***	.24***	.24***	.24***
Effort expectancy			.42***	.42***	.35***
Social influence					
Self-efficacy					.01**

Dependent variable “intention to use e-Health” (1–5). Betas with a p value > = .10 are omitted.

*p value < .10.

**p value < .05.

***p value < .001.

Every block in the regression analysis adding an explanatory variable showed a statistically significant increase in explained variance for performance expectancy, effort expectance, and self-efficacy, although the increase of 1% after adding Internet experience (ease of use) was only marginal. Social influence, though correlated with intention to use (Pearson r = .25, p < .001) in the bivariate analysis, did not have any additional explanatory power in the regression.

Hence, the intention to use e-Health applications was mainly explained by performance expectancy, effort expectancy, and self-efficacy. No effect for social environment was found.

## Discussion

In general, community-dwelling older people are open-minded towards e-Health. If offered an e-Health application, around two-thirds (63%) definitely or probably intend to use it. People with a lower level of education were less likely to intend using e-Health. Also, generally women and respondents in the older age categories showed less intention to use e-Health, but further subgroup analyses showed that a low level of education was the most important impeding factor. We also found that community-dwelling older people were most positive about the effort (or lack of effort) involved in using e-Health. More doubts existed about the benefits of e-Health applications. Only a minority expected that such applications would be useful and that e-Health would provide a pleasant way to interact with care professionals.

In addition, we found that people who strongly believed that using e-Health would help them (performance expectancy) and that e-Health would be easy to use (effort expectancy), were more inclined to use Internet applications in the future. On the other hand, people who did not believe in the possible advantages of e-Health or believed that it would be difficult to use were less inclined to use e-Health. In addition, the belief in one’s own Internet skills (self-efficacy) was related to intention to use whereas social influence was not. Other studies also found that performance expectancy is the most important determinant of intention to use [22].

### People who do not use the Internet or believe use will be difficult

Nearly a quarter of the older people had either never used the Internet (8%) or believed that the Internet is difficult to use (15%). People who believed that using the Internet was difficult were found to be less inclined to use e-Health applications in the future. We also found that they were less convinced of the benefits of e-Health and that they expected more difficulties with using e-Health applications. This group requires special attention to improve their readiness to use e-Health. They need easy-to-use applications and extra guidance on how to use the applications. While communication can influence performance expectancy, experience is needed to change the belief that it is easy to use. Experimental research showed that a brief use of an e-Health application can decrease the expected effort [23,24]. So it is expected that offering older people an opportunity to get acquainted with an e-Health application will increase their intention to use e-Health in future. On the other hand the results show that a significant group among older people will probably not be able or willing to use e-Health and will need supplementary support.

In the long run there will be new generations of elderly people who have grown up with the Internet. If people begin using alternative forms of health-care delivery earlier in life, they may be more likely to continue being users when they develop one or more chronic illnesses later on in life. Longitudinal research will elucidate whether healthy people who have already used the Internet for their care, for instance to communicate with their GP, are more likely to use e-Health when they need more care.

### The role of social influence

Although significant others such as family and friends are found to influence the intention to use e-Health [10], we found no additional value in explaining the intention to use e-Health after the beliefs about the utility (performance expectancy) and ease of use (effort expectancy) had been taken into account. This might be due to the way we operationalized social influence. We asked what significant others would do and not what these significant others wanted the respondent to do. Venkatesh et al. [12] stated that social influence only plays a role in a mandatory context. We hypothesize that as people become increasingly dependent on health-care services, they will also become more open to the influence of others.

### Strengths and limitations

A strength of this study is that a large, nationally representative sample of community-dwelling older people was used. A limitation is that people with a low level of education are underrepresented in the panel and therefore in this study. The same is true for people with very low functional health literacy, who are less willing to participate in a panel that uses written questionnaires and are found to be less likely to seek and use health information [25]. The estimated intention to use internet applications among older people will therefore be too optimistic.

Another limitation is the broad range of existing e-Health services, which are also still rapidly evolving. E-Health services mean different things to different people and services will often be different to what people expected of them once they are implemented.

### Theoretical and practical implications

Further research is needed on facilitating and impeding factors that supplement the UTAUT concepts. Venkatesh et al. proposed an extension of UTAUT for studying acceptance in consumers and added three constructs to UTAUT: hedonistic motivation (the pleasure derived from using the technology), price value (the costs and quality), and the habit of already using the technology [26]. There is also empirical evidence for other facilitating conditions that play a role in older people’s adoption of e-Health, such as the costs of Internet applications [10,27,28], technical support [27], and health status or health-care needs [10,29,30], and the relationship with the health professional [28,31,32].

This study has practical implications in particular for policy makers and care professionals who strive for more technology within health care for community-dwelling older people. Special attention should be paid to people who do not have any experience with the Internet and those who show less intention of using e-Health applications. They need very easy-to-use applications, to be used preferably on devices that they are already accustomed to. It is important that a new application functions well and has no bugs. However obvious, this is often not the case. Research showed that malfunctioning is one of the most frequently occurring barriers when introducing new technologies [33]. Individualized information and consultation, and opportunities to try applications out may boost acceptance and use. Our research also underlines the importance of motivating community-dwelling older people by showing the potential beneficial effects of e-Health applications. Nursing staff can play a role, but they must themselves be convinced of the potential benefits for the patient. It has been found that when nursing staff thought the patient would benefit from the new technology, the nursing staff themselves were more willing to actually use it. The opposite was also found: when the anticipated benefits for the patient were thought to be low or unclear, this impeded the introduction [33]. So when introducing new e-Health applications, professionals must also believe in the benefits of a new technology.

## Conclusions

Older people generally are open minded towards e-Health. However, not all of them will easily use it. Nearly a quarter of the older people will encounter difficulties with e-Health, either because the never used Internet before or believe that Internet is difficult to use. Special attention should be paid to women, people with less education, and people without Internet experience. Acceptance of e-Health can be increased by informing people about the potential benefits of e-Health and letting them practice with the application.

## Additional file

Additional file 1:
**Questionnaire.** Housing and care now and in the future. Part F: Care through the Internet.
