# Supplementary material for: Determinants of the intention to use e-Health by community dwelling older people
Source: BMC Health Serv Res. 2015 Mar 15;15:103. doi: 10.1186/s12913-015-0765-8 (PMC4364096; doi:10.1186/s12913-015-0765-8)
Supplement: Additional file 1: — Questionnaire. Housing and care now and in the future. Part F: Care through the Internet. [file 12913_2015_765_MOESM1_ESM.docx]

registration number


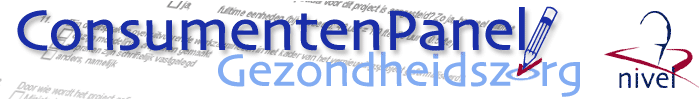


NIVEL

Nederlands instituut

voor onderzoek van de

gezondheidszorg

Freepost 4026

3500 VB Utrecht

Questionnaire

Housing and care now and in the future

| **F Care through the Internet** |
| --- |

In the future you will increasingly be able to use the Internet for your care. For instance, it will be possible to use the Internet for:

- Making an appointment with a health-care professional;

- Asking a health-care professional a question;

- Getting treatment or support via telecare from a health-care professional;

- Measuring your weight, blood pressure or blood sugar level, for example, at home and sending the measurement to your health-care professional.

The Internet can be used on all kind of devices, such as a computer, tablet or smartphone. Besides these Internet contacts, you would still have face-to-face contacts with the health-care professional.

1. Do you think you would use one of the above-mentioned Internet applications in the future if you were offered the opportunity?

- yes, definitely
- yes, probably
- I don’t know yet
- no, probably not
- no, definitely not

1. Do you think you would use one of the above-mentioned Internet applications in the future if it could help you to live independently for longer?

- yes, definitely
- yes, probably
- I don’t know yet
- no, probably not
- no, definitely not

1. How easy or difficult do you find it to use the Internet?

- very difficult
- difficult
- neutral
- easy
- very easy
- I don’t know; I don’t use the Internet

1. What do you expect from the Internet if you need (more) care in the future? To what extent do you agree with the following statements?

| **Contacting health- care professionals via the Internet …** | *strongly disagree* | *disagree* | *agree* | *strongly agree* | *I don’t know* |
| --- | --- | --- | --- | --- | --- |
| a. is easy to do | ❑ | ❑ | ❑ | ❑ | ❑ |
| b. is easy to learn | ❑ | ❑ | ❑ | ❑ | ❑ |
| c. is reliable | ❑ | ❑ | ❑ | ❑ | ❑ |
| d. works well | ❑ | ❑ | ❑ | ❑ | ❑ |
| e. is a pleasant way to interact with health-care professionals | ❑ | ❑ | ❑ | ❑ | ❑ |
| f. fits easily into my daily routine | ❑ | ❑ | ❑ | ❑ | ❑ |
| g. is something my family or friends would like to do | ❑ | ❑ | ❑ | ❑ | ❑ |
| h. makes it easier to contact a health-care professional when I want | ❑ | ❑ | ❑ | ❑ | ❑ |
| i. enables me to live independently for longer | ❑ | ❑ | ❑ | ❑ | ❑ |
